# Developing a patient care pathway for emotional support around the point of multiple sclerosis diagnosis: A stakeholder engagement study

**DOI:** 10.1111/hex.13711

**Published:** 2023-01-23

**Authors:** Tierney Tindall, Gogem Topcu, Shirley Thomas, Clare Bale, Nikos Evangelou, Avril Drummond, Roshan das Nair

**Affiliations:** ^1^ Mental Health and Clinical Neurosciences, School of Medicine University of Nottingham Nottingham UK; ^2^ Injury, Inflammation and Recovery Sciences, School of Medicine University of Nottingham Nottingham UK; ^3^ Nottingham Multiple Sclerosis Patient and Public Involvement Group Nottingham UK; ^4^ Department of Neurology Nottingham University Hospitals NHS Trust Nottingham UK; ^5^ Rehabilitation Research Group, School of Health Sciences University of Nottingham Nottingham UK; ^6^ Institute of Mental Health Nottinghamshire Healthcare NHS Trust Nottingham UK; ^7^ Health Division, SINTEF Trondheim Norway; ^8^ Present address: Department of Neuroscience, Psychology and Behaviour University of Leicester Leicester UK

**Keywords:** diagnosis, emotional support, multiple sclerosis, peer support, qualitative, stakeholder engagement

## Abstract

**Background:**

Diagnosing multiple sclerosis (MS) can be a lengthy process, which can negatively affect psychological well‐being, condition management, and future engagement with health services. Therefore, providing timely and appropriate emotional support may improve adjustment and health outcomes.

**Purpose:**

To develop a patient care pathway for providing emotional support around the point of diagnosing MS, and to explore potential barriers and facilitators to delivery and implementation.

**Methods:**

Focus groups were conducted with 26 stakeholders, including 16 people living with MS, 5 carers/family members and 5 professionals working with people living with MS (3 MS nurses, 1 psychiatrist, and 1 charity staff member). Discussions were audio‐recorded, transcribed verbatim and analyzed using framework analysis.

**Results:**

Participants suggested that a patient care pathway should include comprehensive information provision as a part of emotional support at diagnosis, and follow‐up sessions with a healthcare professional. Barriers including increasing staff workloads and financial costs to health services were acknowledged, thus participants suggested including peer support workers to deliver additional emotional support. All participants agreed that elements of a care pathway and embedded interventions should be individually tailored, yet provided within a standardized system to ensure accessibility.

**Conclusions:**

A patient care pathway was developed with stakeholders, which included an embedded MS Nurse support intervention supplemented with peer support sessions. Participants suggested that the pathway should be delivered within a standardized system to ensure equity of service provision across the country.

**Patient or Public Contribution:**

This research was conceptualized and designed collaboratively with Nottingham Multiple Sclerosis Patient and Public Involvement and Engagement (PPIE) group members. One member is a co‐author and was actively involved in every key stage of the research process, including co‐design of the pathway and research protocol, data collection (including presenting to participants and moderating group discussions), analysis and write‐up. Authors consulted with PPIE members at two meetings (9 and 11 PPIE attendees per meeting) where they gave feedback on the research design, findings and the resulting pathway. People living with MS and carers of people with MS were included in the focus groups as participants.

## 1 INTRODUCTION

Multiple sclerosis (MS) is a chronic neurological condition, often diagnosed in mid‐adulthood, and is the most common cause of nontraumatic neurological disability in working‐age adults.^1^ Symptoms of MS, which may include ‘visible’ (e.g., dexterity and mobility problems) and ‘invisible’ symptoms (e.g., fatigue, cognitive problems),^2^ result from inflammation and demyelination of the central nervous system.^3^, ^4^ An estimated 2.8 million people live with MS worldwide, with around 107,000 new diagnoses each year^1^—a frequency that emphasizes the importance of delivering diagnostic news which meets the needs of an increasing clinical population. However, as there is no single, simple diagnostic test, diagnosing MS can be a lengthy process for individuals, which can cause confusion, relief, distress, and frustration.^5^, ^6^, ^7^

The general well‐being of people with MS can be impacted by how they adapt to their changing health circumstances.^8^ Psychological adjustment refers to the process of adapting to circumstances such as chronic disease and associated treatment,^9^ whereby the individual aims to maintain equilibrium between competing environmental demands and the resulting stress. The way in which the period surrounding diagnosis is managed may determine how successfully a person adjusts to MS, influencing future perceptions of their condition, and may affect subsequent engagement with services.^6^ The prediagnosis period in which symptoms are investigated can be particularly distressing due to the perceived uncertainty while awaiting diagnosis.^6^ Qualitative evidence shows feelings of being misunderstood before the legitimization of individuals' condition by confirmed diagnosis, such that diagnosis produced feelings of devastation which conflicted with relief at being able to explain symptoms that were previously disbelieved by others.^6^ Therefore, ensuring that people receive comprehensive information and support from the beginning of their lifelong MS journey may be crucial to facilitating positive psychological adjustment, while improving treatment outcomes and long‐term management.

A recent meta‐synthesis showed that many newly diagnosed people with MS had unmet emotional and informational needs during their diagnosis period.^10^ In a qualitative study of experiences of adjusting to early‐stage MS, many participants described feeling fear at being given a diagnosis and feeling overwhelmed by thoughts of impending doom.^5^ However, they also felt that seeking positive, optimistic information increased their ability to accept the diagnosis and their perceived control over MS, while social support was regarded as critical for their adjustment.^5^ Moreover, other qualitative research has suggested that providing adequate information about their condition and its treatment options at diagnosis may reduce feelings of anxiety and uncertainty.^6^ This suggests that providing positively framed information coupled with social support may be key to facilitating successful psychological adjustment and that informational support is considered a part of emotional support.^5^, ^6^ Similarly, a recent metareview of systematic reviews on adjustment to MS suggested that professional support, information provision, continuity of care and peer support are factors throughout the diagnostic process which were linked to better psychological adjustment.^11^

The value of providing accessible information, suitable advice and support at diagnosis is well recognized.^12^, ^13^ However, current literature demonstrates that poor support and information provision has continued for people with MS throughout their diagnosis period^10^, ^11^ and should be part of emotional support.^5^, ^6^ A survey of people with MS in the United Kingdom identified that information provision at diagnosis was inconsistent.^14^ Inadequate information provision has persisted over time and appears to be a common issue across Europe.^6^, ^7^, ^10^, ^11^, ^15^ Findings from a meta‐review showed that there are no adequate emotional support interventions that specifically target individuals newly diagnosed with MS.^11^ Most resources dealt with providing information about MS (its causes, symptoms and treatment options), rather than broader emotional support including advice around living with MS. Furthermore, there are no established care pathways in the United Kingdom that include emotional support around MS diagnosis, and no referral systems to seamlessly incorporate wider ‘third‐sector’ or charity‐based support resources or services.^10^

The healthcare charity sector is an important source of support for many people with specific health issues. In the United Kingdom, the MS Society (www.mssociety.org.uk) and MS Trust (www.mstrust.org.uk) are trusted resources for many people with MS. Shift.MS (www.shift.ms) offers users peer support through its social network to connect with others with MS, helping people to make sense of their diagnosis and adapt to life with MS. These charities and support groups serve a useful adjuvant to the standard care patients receive from the National Health Service (NHS; e.g., providing emotional and social support) and in some cases, offer support that no one else provides (e.g., help with insurance, support for carers, social connection). In the UK context, however, the NHS and the charity providers for people with MS are somewhat disjointed, resulting in patients not receiving the best care they can receive. To address this gap, we aimed to co‐construct a care pathway to provide emotional support to people around the point of MS diagnosis that linked NHS services with those provided by the charity sector.

**Figure 1:**
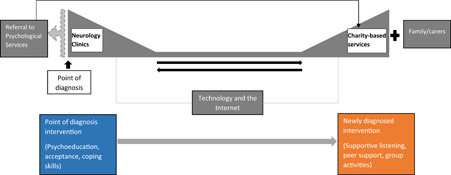
Initial care pathway around the point of diagnosis of MS. ‘Point of diagnosis’ refers to prediagnosis when investigations are underway or when diagnostic news is given to the patient while ‘newly diagnosed’ refers to the following period (up to five years, as per PPIE comments suggesting that uncertainty following diagnosis can last this long). The top part of the diagram (grey) demonstrates a need for a timely referral system to charity‐based services for bridging the gap between the clinic and the charities around the time of the diagnosis and indicates who could provide the care and where it could be provided. The ‘Technology and the Internet’ section describes the medium by which emotional support could be provided in the interim. This would serve as a link between clinics and charity‐based services, and facilitate the transition of support which is provided by clinics to charity‐based services (denoted by the arrows), by offering relevant, accessible and reliable online information and ongoing online support. Referral to Psychological Services could provide individual support during the point of diagnosis, and Psychological Services could also link service users to charity‐based services. Support from Family/carers is intended to supplement support from MS charities, as per suggestions from PPIE consultation. The bottom two boxes describe when particular types of emotional support could be provided across time. MS, multiple sclerosis; PPIE, Patient and Public Involvement and Engagement.

The importance of theory in developing and evaluating complex interventions is well established.^16^ Here, we propose an initial pathway that depicts the theoretical framework for providing emotional support around the point of MS diagnosis (Figure 1). This initial pathway was informed by reviews of literature,^10^, ^11^ pertinent theory,^17^ Patient and Public Involvement and Engagement (PPIE), clinical experience and service realities. It proposes a timely referral system to charity‐based services for bridging the gap between services the clinic and relevant charities can provide around the time of the diagnosis. However, this pathway is preliminary and needs further exploration to determine its utility and acceptability. Moreover, exploring the views of key stakeholders enables the production of experience‐based co‐developed interventions that empower and engage service users.^18^, ^19^

This study aimed to develop a patient care pathway to provide emotional support around the point of MS diagnosis (i.e., prediagnosis when investigations are underway when the diagnosis is given, and in the weeks postdiagnosis). The secondary aim was to explore potential barriers and facilitators to the delivery and implementation of this pathway. We followed a person‐centred,^19^ experience‐based co‐design approach^18^ allowing key stakeholders (i.e., service users and service providers) to inform pathway design *collaboratively* through group discussions, to identify sustainable changes that meet patients' needs. The present study is part of a wider research project aiming to develop and evaluate an intervention to support individuals around MS diagnosis (Providing Emotional Support around the Point of MS Diagnosis—PrEliMS Study^20^).

## 2 METHOD

To ensure the quality of reporting and transparency, we followed the consolidated criteria for reporting qualitative studies (COREQ) and the reporting recommendations for qualitative methods in communication and patient education research.^21^, ^22^

### 2.1 Design

**Figure 2:**
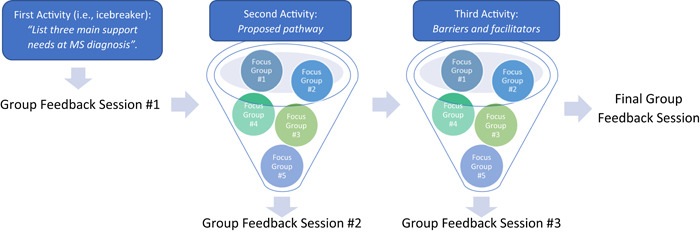
Study design. MS, multiple sclerosis.

A qualitative, multistakeholder engagement study design was used to gain different perspectives from key stakeholders. The engagement involved consulting with stakeholders through different research activities. This consisted of one activity and two discussions, each addressing a different open question, across five individual focus groups and whole‐group feedback sessions following each discussion (Figure 2). All stakeholders participated in the focus groups, and one representative from each group summarized their groups' reflections within whole‐group feedback.

### 2.2 Participants

We refer to stakeholders as individuals targeted by the proposed pathway or involved in its development and/or delivery. Inclusion criteria for each stakeholder category are provided in Table 1. All participants were required to be above 18 years, able to give informed consent and communicate in English. People with MS and carers/family members were recruited through MS charities, local MS support groups and from our local PPIE database. Health professionals and charity staff were recruited through charities and professional networks of the research team. Participants were a self‐selected convenience sample, weighted towards people living with MS, in line with the experience‐based co‐design approach.^18^

**Table 1:** Inclusion criteria for each stakeholder group

Stakeholder group	Inclusion criteria
People with MS	People referred for possible MS diagnosis (i.e., the period just before receiving a formal diagnosis of MS by a neurologist up to 5 years), or newly diagnosed with MS (up to 5 years postdiagnosis) or have been diagnosed longer than 5 years ago who could comment on their past experiences. We used a 5‐year period because, for some people with MS, the uncertainty around diagnosis lasted for this long, and our PPIE group felt that it was important to capture those who were within this period.
Carers/family members	Relative/carer of a person with MS. Carer is defined as a relative or friend providing informal care for someone with MS.
Health professionals	Clinicians (e.g., neurologists, MS nurses, psychologists, occupational therapists) working with people with MS.
MS charity staff/volunteers	People working or volunteering within MS charities.

Abbreviations: MS, multiple sclerosis; PPIE, Patient and Public Involvement and Engagement.

### 2.3 Procedure

Participants were invited to a local community centre to participate in a day‐long event involving planning a patient care pathway for people with MS at the point of diagnosis. Before discussions, informed consent and relevant demographic information were obtained from all participants. The agenda for the day was co‐created with our research team, which included a person with MS. Five semistructured focus groups of five to six participants (representing different stakeholder categories) sat at tables arranged Cabaret‐style for ease of discussions, moderated by five research‐active clinicians (a consultant neurologist, occupational therapist, clinical psychologist and two trainee psychologists; one moderator per focus group).

The event began with a presentation of what is already known about emotional support at MS diagnosis and the study aims. The first activity (‘What do you think are the three main things that need to be in place to support emotional wellbeing around the point of MS diagnosis?’) was used as an icebreaker. Participants wrote ideas on little sticky‐notes individually, which guided reflections for the second discussion. Participants were provided with a diagram outlining a proposed care pathway designed based on literature, theory, clinical experience of research‐active clinicians and a consultation meeting with nine PPIE members recruited from the Nottingham Multiple Sclerosis Patient and Public Involvement groups (Figure 1). Participants were invited to use the model to initiate the second discussion, exploring participants' suggestions for elements to add/remove from the pathway. Moderators used open‐ended questions to probe for suggested changes, additions, the timing of various activities, who might deliver support and how/when it might be offered. The third discussion considered possible barriers and facilitators to delivery and implementation. After each round of discussion in focus groups, lasting approximately 45 min each, participants engaged in 30‐minute feedback sessions with the whole group, led by a PPIE member. Finally, participants were invited to make additional comments towards the end of the discussions and were debriefed.

### 2.4 Data analysis

Discussions were audio‐recorded and transcribed verbatim, omitting participant‐identifiable information and analysed using framework analysis.^23^, ^24^ The analysis primarily applied a deductive‐inductive approach to address our research aims. Thus, we anticipated that themes such as information, peer support and individualization would be relevant due to their prevalence in the extant literature (i.e., the deductive approach). The data were also examined inductively to allow unexpected responses or new insights.^23^

Transcripts were coded on NVivo (Version 12) by the first author, to classify the data and allow for systematic comparisons across discussions. A working analytical framework was developed after coding one focus group transcript for both discussions and whole group feedback sessions, reviewed by two other authors. Codes were grouped into categories, with new codes added as subsequent transcripts were indexed by the first author. Discussions with the rest of the research team were held to sense‐check and modify the coding scheme. Any disagreements were resolved by discussion until consensus was reached, or arbitrated if necessary, by the wider research team. Yardley's^25^ evaluative characteristics for good qualitative research were followed to ensure credibility and rigour.

## 3 RESULTS

The stakeholder sample consisted of 16 people with MS (68.75% relapsing‐remitting, 18.75% secondary progressive, 12.5% primary progressive) with an average illness duration of 8.6 years (SD = 6.6); 5 carers/family members of a person with MS (4 partner/spouse; 1 parent) and 5 healthcare professionals and charity staff (3 MS nurses, 1 psychiatrist and 1 MS Society staff member) with a range of 10–33 years experience working with MS (*M* = 17.8; SD = 10.3). Table 2 describes further demographics.

**Table 2:** Participant demographics per stakeholder group

Demographics (*n*, %)	People with MS (*n* = 16; 62%)	Carers/family (*n* = 5; 19%)	Healthcare professionals and charity staff* (*n* = 5; 19%)	Overall** (*n* = 26)
Age				
21–30	1 (6.25%)	0 (0.0%)	0 (0.0%)	1 (4.0%)
31–40	4 (25.0%)	0 (0.0%)	1 (20.0%)	5 (20.0%)
41–50	4 (25.0%)	0 (0.0%)	0 (0.0%)	4 (16.0%)
51–60	3 (18.75%)	1 (20.0%)	2 (40.0%)	6 (24.0%)
60+	4 (25.0%)	4 (80.0%)	1 (20.0%)	9 (36.0%)
Gender				
Man	5 (31.25%)	4 (80.0%)	2 (40.0%)	11 (42.3%)
Woman	11 (68.75%)	1 (20.0%)	3 (60.0%)	15 (57.7%)
Education level				
GCSE	6 (37.5%)	0 (0.0%)		6 (28.6%)
A Level	3 (18.75%)	0 (0.0%)		3 (14.3%)
Degree	4 (25.0%)	4 (80.0%)		8 (38.1%)
Higher degree	3 (18.75%)	1 (20.0%)		4 (19.0%)
Employment status				
Not employed	2 (12.5%)	0 (0.0%)		2 (9.5%)
Employed full‐time	1 (6.25%)	1 (20.0%)		2 (9.5%)
Employed part‐time	4 (25.0%)	1 (20.0%)		5 (23.8%)
Retired	8 (50.0%)	3 (60.0%)		11 (52.4%)
Voluntary part‐time	1 (6.25%)	0 (0.0%)		1 (4.8%)

*One professional did not disclose age. **Education level and employment status information was not collected from healthcare professionals and charity staff.

Following framework analysis, 31 subthemes were interpreted (presented in italics) relating to the development of an emotional support pathway, organized into 5 superordinate categories (Table 3).

**Table 3:** Categories and subthemes identified

Category	Subthemes
Information	Condition management
	Services
	Accuracy and reliability
	Social network and employers
	Practical information
	Overload
	Format
	Cooling off period
	Framing
Individualization	Person‐centred approach
	Toolbox of options
	Patient experience
	Family involvement
	Timing
Standardization	Accessibility
	Postcode lottery
	Technology integrating services
	Evaluation
	Hub
Professional role	Personal attributes
	Duties
	Training
	Value of human connection
	Governance
	Limitations
Voluntary peer support role	Shared experiences
	Benefits to peer supporter
	Savings
	Sustainability
	Befriender

### 3.1 Information

Participants across individual focus groups felt that a package of adequate, relevant and appropriate information should be provided at the point of diagnosis, for example for *condition management* and signposting to *services* and MS charities. Participants agreed that *practical information* regarding responsibilities such as contacting the UK Driver and Vehicle Licensing Agency (which issues drivers' licences) and general advice for daily life should be given to people with MS, and their *social network and employers*. Participants suggested that verbal information should be accompanied by different *formats*, such as hard‐copy booklets to keep and refer to later. Most participants discussed that information should come from a reliable source to avoid receiving inaccurate information (*accuracy and reliability*), as participants in two focus groups opined ‘don't google’ (Groups 1 and 2). Overall, participants felt that information and diagnostic news should be positively framed with hope (*framing*):

I think the biggest thing I could have found out at the start was someone saying, ‘you're not alone, there is support there’ (Woman with MS, Group 2)

However, participants recommended that clinicians should strike a balance between giving sufficient information—which they felt was an important element of emotional support facilitating ongoing psychological adjustment—and avoiding *overload*. One participant stated that they felt bombarded with medical information at diagnosis: ‘You just can't assimilate it’ (Woman with MS, Group 1). To overcome this issue, four focus groups discussed a *cooling off period*, whereby patients should be given time to process their MS diagnosis, for example allowing a 2‐week interval between diagnosis and a follow‐up session with a healthcare professional, such as an MS Nurse.

### 3.2 Individualization

Focus groups elaborated on the *timing* of the pathway's elements, which was also repeated during whole‐group feedback. They felt it should depend on the patient's unique needs and preferences. Furthermore, participants felt that it was essential that a patient care pathway provides an individualized, *person‐centred approach* to account for their personal circumstances, such as *family involvement*, ‘because what suits one person might not [suit] another’ (Carer, Male, Group 3). Participants reflected on their varied *patient experience* and suggested that adjustment is a different process for every person, thus the diagnosis procedure ‘must be fluid’ (Carer, Male, Group 5). Therefore, participants felt that individuals should have access to a *toolbox of options* via professional contact, with alternatives based on need and personal choices, such as topic‐based information or referrals:

keep it as simple as you can, but say ‘here are some things that may be available if you need it’ (Man with MS, Group 2)

### 3.3 Standardization

While participants emphasized the importance of individualization, they agreed that some standardization is required to ensure *accessibility. Postcode lottery* should be avoided, whereby a person's geographical location in the country can determine the service they receive. Therefore, participants suggested using *technology* (e.g., the Internet, videoconferencing) to standardize service delivery throughout the pathway to be accessible for everyone. However, some participants felt that this would not be appropriate for all, arguing that some people avoid the Internet or would benefit more from in‐person sessions rather than telephone calls. Again, this is linked with individualization and applying a person‐centred approach throughout.

To avoid geographical location‐based limitations, participants felt that *integrating services* through a ‘holistic approach’ (Woman with MS, Group 4) would facilitate collaboration between the NHS and other organizations, such as charities, to reach out to people in rural areas with fewer services. For example, participants suggested creating a *hub* to direct patients to different services or professionals. Finally, participants discussed the importance of *evaluation* of the pathway, because ‘when the NHS has got evidence, they're compelled to act’ (MS Nurse, Female, Group 5), to ensure its sustainability from a resource perspective.

### 3.4 Professional role

All focus groups felt strongly that a paid healthcare professional should deliver part of the intervention. Most agreed that this should be an MS Nurse, while a smaller selection of participants wondered whether it would save costs to develop this role for less qualified staff. *Personal attributes* of the professional were discussed, whereby participants described essential qualities such as expertise, experience and understanding of MS: ‘somebody who's got that appropriate knowledge—whoever that is’ (Man with MS, Group 1).

*Duties* of the role included referrals and triaging for emotional support according to clinical need, including an ‘individual assessment of their needs in the first instance’ (MS Nurse, Female, Group 5). Participants agreed that professionals should give patients time to ask questions and listen to concerns at diagnosis, initiating the provision of emotional support. Many felt that this professional should be present at diagnosis, alongside the neurologist delivering the news, as an MS champion and advocate. Groups agreed that the professional should be introduced in person to build a relationship for personalized emotional support, emphasizing the *value of human connection*:

I would have liked there to have been an MS nurse with us, but then took us aside and said like, ‘you've been told [about your MS diagnosis]—what are your questions?’ (Woman with MS, Group 2)

However, *limitations* of the role included the barrier of recruiting and retaining enough staff. Two focus groups discussed the demands of nursing roles, with long hours and large workloads, which would likely increase with extra responsibilities accompanying delivering a new intervention within the pathway. To overcome these barriers, participants felt the role would need appropriate *governance* to set parameters for the role, with *training* and supervision to quality‐check support, hence its requirement for remuneration and professional accountability:

if you're properly trained by a body, and are affiliated to do it, then you're alright. It is protection. (Man with MS, Group 3)

Furthermore, participants proposed outsourcing and delegating duties to a voluntary support worker role, to deliver further aspects of emotional support via a ‘parallel service’ (Woman with MS, Group 5), offsetting the burden of nurses' long hours and increasing workload.

### 3.5 Voluntary peer support role

Some participants across groups felt that peer supporters could be paid with ‘savings that these programmes will achieve’ (Woman with MS, Group 4), facilitating their remuneration, yet most referred to this role as voluntary to save costs for the NHS. In addition, groups suggested that *savings* could be made across wider NHS capacity. Developing a role for peers also living with MS could mitigate the barrier of not having enough time or staff, as another participant noted, ‘time is precious for medical professionals in the NHS’ (MS Nurse, Female, Group 2), which may not be an issue for volunteers by virtue of them volunteering time. Contrastingly, the *sustainability* of voluntary roles was questioned:

When you volunteer, sometimes you can get dragged into doing so much that actually it's a detriment to yourselves. (Charity staff, Female, Group 3)

Therefore, finding appropriate volunteers who are committed to the intervention's duration with each patient was presented as another barrier, which participants suggested could be mitigated by training and ongoing support. A further facilitating factor was discussed: *benefits to peer supporter*. Participants felt that people with MS might find this role fulfilling and thus remain enthusiastic and committed to ‘giving something back’ (Man with MS, Group 4), obviating the need for payment, particularly for people who might be transitioning from employment to retirement:

if they had to give up work because of their condition, this might be a way that they can still feel valuable. (Man with MS, Group 3)

Groups unanimously agreed that the crucial facilitator of peer support within a pathway was the contribution of *shared experiences*. Many participants felt that individuals living with MS would be more relatable and credible, as most clinicians cannot understand the subjective experience of MS. Therefore, sharing relevant experiences may help patients to problem‐solve, which many felt was vital for emotional support. Moreover, participants suggested that the peer supporter could act as a *befriender* to listen and empathize because they felt that sometimes patients need more emotional support than a healthcare professional can provide. Therefore, peers ‘on the same wavelength’ (Woman with MS, Group 5) could be instrumental during the process of adjustment around the point of diagnosis.

**Figure 3:**
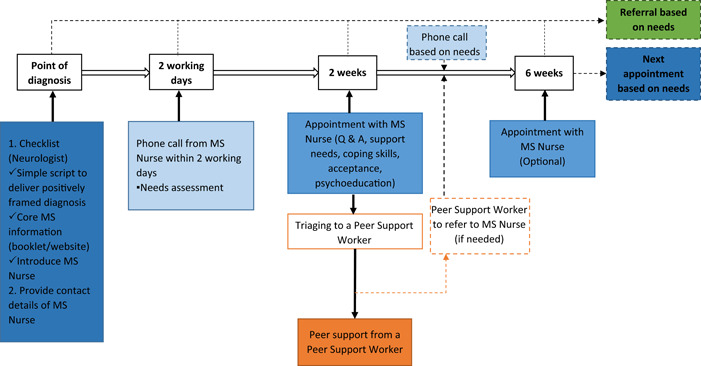
A patient care pathway for emotional support provision around MS diagnosis, co‐constructed with stakeholders. MS, multiple sclerosis.

A patient care pathway was subsequently created by the research‐active clinicians present during the stakeholder engagement discussions, in light of participants' reflections. This was then presented to a group of seven people with MS and four carers, who helped us to refine the pathway. Figure 3 depicts the co‐constructed pathway.

## 4 DISCUSSION

Stakeholder focus groups and whole‐group discussions informed the development of a blueprint for a co‐constructed ‘point of MS diagnosis’ patient care pathway to provide emotional support around the point of diagnosing MS (Figure 3). Many participants agreed that the proposed pathway should involve MS Nurses at the forefront, who should be introduced to the patient early during discussions about the diagnosis. Groups also agreed that adequate, appropriately delivered information is a key element of emotional support, equipping patients with reliable advice for dealing with practical issues, which should be positively framed to instil hope. The addition of peer support volunteers to assist during the pathway was suggested and accepted by many participants. This may also improve the health and well‐being of the volunteers themselves.^26^ However, some participants worried about the role's sustainability, whereby volunteers may not remain committed without payment. Furthermore, there are valid debates about volunteer remuneration^27^ that are beyond the scope of this paper, but nonetheless need to be considered.

In the United Kingdom, our MS services are embedded within a nationalized healthcare system (NHS) funded by the state.^28^ While this affords equality of care, it poses certain funding and service constraints (with overstretched and understaffed services). This is one of the reasons why we explored who would be best placed to offer emotional support services and considered the potential involvement of a volunteer workforce. Thus, issues of sustainability could be mitigated by offering patients combined input from MS Nurses and peer supporters. As such, this could offset barriers regarding limited resources, such as time and funding, while containing the voluntary role within certain boundaries to ensure that the volunteers are not overburdened or work outside of their expertise. Indeed, while participants suggested that retirees with MS may be well‐suited, recruitment and retention may be difficult, especially if volunteers are limited by the progression of MS symptoms. Alternatively, there have been several models of such peer‐support work in the mental health arena, which have shown great value,^29^, ^30^, ^31^ and the NHS has created job descriptions that have been mapped onto NHS pay scales. Therefore, such models could be adopted in MS services.

Our findings support the existing research literature, particularly around the timing of commencing the pathway and providing the embedded support intervention. Participants felt that after delivering news of the diagnosis, further support should be delayed to allow the individual time to process the diagnosis. Moreover, participants felt they needed timely information regarding reliable sources of information. This echoes the line of argument resulting from a recent meta‐synthesis.^10^ Others also recommend that professionals delivering diagnoses of neurological conditions (including MS) should assess and respond to patients' information preferences with empathy and provide more time for questions,^32^ which our findings support. Indeed, while individuals may have different information needs due to personal circumstances, several studies have highlighted inadequate information provided.^6^, ^14^, ^33^, ^34^, ^35^ Reflecting Miller's^36^ theory of coping with stress‐provoking situations, a study proposed that people with MS could be categorized into ‘monitors’, who seek information to manage their MS, or ‘blunters’, who avoid information as they believe it increases anxiety.^33^ Another study found that a significant proportion of respondents declined further advice (‘blunters’), suggesting that the impact and severity of one's MS affects their need for information.^14^ Our results also support the provision of comprehensive, individualized information which may benefit longer‐term emotional adjustment. Also, our findings urge that information provided must be given to individuals at the time they need it, ensuring they have time to digest new information to improve experiences around diagnosis. Nevertheless, an examination regarding the type of information people with MS find helpful/unhelpful and how it might be incorporated as part of emotional support would improve the usefulness of the findings. Additionally, further qualitative research could explore how to support those who specifically undergo a prolonged period of diagnostic investigations during prediagnosis, as this may present unique challenges. The present study focused on the development of the patient care pathway around MS diagnosis *in general*, including prediagnosis, diagnosis and the immediate postdiagnosis period.

Strengthening the study, we followed Yardley's^25^ guiding principles for good quality qualitative research: context sensitivity, commitment and rigour, transparency and coherence and impact and importance. Commitment considers topic engagement and data immersion which is supported by our evidence‐based approach, strengthening context sensitivity. To ensure the rigour of the findings, different stakeholder groups collaborated to achieve multilayered insights and whole‐group feedback triangulated responses from focus group discussions.^25^ While at least two researchers analysing data separately before reaching a consensus has been recommended,^37^ the concept of interrater reliability in qualitative research has been regarded as meaningless and cannot exclude subjective interpretations.^25^ However, two coders may have further improved rigour and transparency to reach a consensus without the need to calculate interrater reliability. In the interest of transparency, the researcher responsible for the initial coding of transcripts was absent from the event, thus missing an opportunity to become immersed in the process and begin familiarization during data collection.^23^ To mitigate this limitation, they listened to recordings multiple times to aid familiarization and received input from team members present at the event when developing the coding scheme to enhance collaboration during analysis. Considering coherence, adopting framework analysis based on a person‐based co‐design was appropriate for our aims, as framework analysis is particularly suitable when study aims are clearly defined at the onset.^23^

Furthermore, the present study aimed to maximize impact and utility^25^ by adhering to the Medical Research Council's guidance for complex intervention implementation, and by developing relevant theory from literature and stakeholder engagement before commencing the lengthy evaluation process.^16^ Thus, by identifying important active ingredients based on the person‐ and experience‐based co‐design approaches, the resulting pathway and embedded support intervention are more likely to be sustainable whilst meeting patients' needs, maximizing the likelihood of acceptability during subsequent evaluation within a full feasibility trial.^19^ Moreover, context‐sensitivity is increased by eliciting perspectives of different stakeholder groups, illuminating potential barriers and facilitators to inform implementation within healthcare settings.^25^ While research‐active clinicians acting as moderators in the focus groups may have biased discussions (e.g., reflecting professional disciplines), all have varied clinical backgrounds offering diverse perspectives.

Recognizing the need for further emotional support around MS diagnosis, we have developed a co‐constructed patient care pathway for emotional support provision around MS diagnosis via multistakeholder engagement. Our stakeholders felt that information provision coupled with emotional and social support (individualized MS Nurse support, peer support) would improve adjustment to diagnosis, reflecting prior research.^5^, ^6^, ^7^, ^10^, ^11^, ^12^, ^13^, ^15^ Participants also suggested that emotional support should be delivered within a standardized system, including those who may miss out due to sparse services in their location, and/or have difficulties accessing technology and the Internet. However, barriers were noted, including financial costs to the NHS and demand on nurses' limited time, which could be mitigated by enlisting peer support workers to facilitate ongoing emotional support.

As part of the PrEliMS programme, a point of diagnosis intervention was developed using this pathway and is currently being tested in a feasibility trial, and a definitive trial evaluating the clinical and cost‐effectiveness of this pathway is planned. Future research could consider whether matching patient participants to peer supporters (e.g., across specific demographic variables) would be beneficial, as patient participants may have different priorities across life stages (such as work, raising children and retirement), and research should explore how the peer support worker role in mental health services can be adapted for MS services.

## AUTHOR CONTRIBUTIONS

**Tierney Tindall**: Data curation; formal analysis; methodology; writing – original draft; writing – review and editing. **Gogem Topcu**: Conceptualization; methodology; investigation; formal analysis; writing – original draft; writing – review and editing; supervision; project administration; funding acquisition. **Shirley Thomas**: Methodology; formal analysis; writing – review and editing; supervision. **Clare Bale**: Conceptualization; investigation; writing – review and editing. **Nikos Evangelou**: Conceptualization; investigation; writing – review and editing; funding acquisition. **Avril Drummond**: Conceptualization; investigation; writing – review and editing; funding acquisition. **Roshan das Nair**: Conceptualization; investigation; writing – review and editing; funding acquisition.

## CONFLICTS OF INTEREST

R. d. N. is the Chair of the NIHR Research for Patient Benefit East Midlands Research Advisory Committee; he has received funding to prepare and deliver lectures on cognitive rehabilitation in multiple sclerosis from Novartis and Biogen. N. E. is a member of the advisory board for Biogen, Merck, Novartis and Roche; he has received grant income from the MS Society, MRC, PCORI and NIHR. The remaining authors declare no conflict of interest.

## ETHICS STATEMENT

Ethical approval was obtained from the University of Nottingham Faculty of Medicine and Health Sciences Ethics Committee (193‐801). Participants gave informed consent to participate and for anonymized results to be disseminated including through publication. We confirm all personal identifiers have been removed or disguised so the patient/person(s) described are not identifiable and cannot be identified through the details of the story.

## Data Availability

The data that support the findings of this study are available on request from the corresponding author. The data are not publicly available due to privacy or ethical restrictions.
